# Clinical outcomes of contemporary lateral augmentation techniques in primary ACL reconstruction: a systematic review and meta-analysis

**DOI:** 10.1186/s40634-021-00368-5

**Published:** 2021-08-12

**Authors:** Lucas Beckers, Thiago Vivacqua, Andrew D. Firth, Alan M. J. Getgood

**Affiliations:** 1 Department of Orthopedic Surgery, Fowler Kennedy Sport Medicine Clinic, University of Western Ontario, 3M Centre, 1151 Richmond Street, London, ON N6A 3K7 Canada; 2 Health and Rehabilitation Sciences, Faculty of Health Sciences, University of Western Ontario, 3M Centre, 1151 Richmond Street, London, ON N6A 3K7 Canada

**Keywords:** ACL, Anterior cruciate ligament reconstruction, Systematic review, Meta-analysis, Lateral extra-articular tenodesis, Anterolateral ligament, Pivot Shift, Rotational instability

## Abstract

**Purpose:**

The purpose of this investigation was to systematically review the contemporary literature to determine if a lateral augmentation (LA) added to an Anterior Cruciate Ligament Reconstruction (ACLR) provides better clinical and patient reported outcomes compared to an isolated ACLR.

**Methods:**

A systematic review and meta-analysis was performed according to the Preferred Reporting Items for Systematic reviews and Meta-analyses (PRISMA) criteria. Two authors independently conducted an electronic search using MEDLINE® and Embase® on February 6^th^, 2021 for level I-III randomized controlled trials (RCT) and prospective cohort studies without randomization, published after 2012 and with a minimum of two year follow-up. Publications were included when they reported on the objective knee stability examination, patient reported outcome scores, return to sports or graft rupture rate of any type of primary, isolated ACLR compared to ACLR combined with any type of LA.

**Results:**

A total of 11 studies that reported on a combined total of 1892 unique patients were eligible for data extraction, including five RCTs and six prospective cohort studies. In 6 studies, an Anterolateral Ligament reconstruction (ALLR) was the LA of choice, while the 5 other publications used different types of Lateral Extra-articular Tenodesis (LET). A significant reduction in graft ruptures was found in patients treated with ACLR + LA (3%) compared to isolated ACLR (12%). Rotational laxity was significantly higher in isolated ACLR (14%) compared to ACLR + LA (6%). Addition of a LA reduced anterior translation when assessed via instrumented laxity testing. No significant difference was found in the patient reported outcome scores (IKDC and Tegner) between both patient groups, except for the Lysholm Score which was significant in favour of the ACLR + LA group.

**Conclusion:**

Combination of a primary ACLR with a LA can significantly reduce the risk of graft rupture and provide better rotatory stability, without jeopardizing patient reported outcomes.

**Level of evidence:**

Level III, Systematic Review of Level I, II and III studies.

## Introduction

One of the major ‘hot topics’ in Orthopaedic Sports Medicine in the past decade has been the identification and anatomical description of the anterolateral ligament (ALL) [5, 59]. An important reason for the extensive attention to the 'rediscovery' of this structure was its assumed role in the rotatory stabilization of the anterior cruciate ligament (ACL) injured knee [2, 5, 42, 61]. Along with the ALL’s recognition, more emphasis was subsequently placed upon the anterolateral complex (ALC) [12]. This interrelated group of structures on the lateral side of the knee, including the superficial and deep iliotibial band (ITB) with its related capsulo-osseous layer, and the ALL [5, 40, 59] has been proven to assist in the control of the rotatory laxity of the knee [11, 12, 23, 29]. Subsequently, augmentations of the ALC have been considered by some as a breakthrough in the attempt to enhance the survival and outcome of ACL reconstruction (ACLR) [30, 31]. This increased interest has resulted in a plethora of publications on several aspects of the ALC, mainly addressing the ALL. However, contradictory data on the role and the necessity for an ALC repair/augmentation in the setting of primary ACL injured knees resulted in a divergent standpoint regarding this additional procedure in the Orthopaedic sports community [16, 34, 43, 46]. This has been amplified by limited high-quality clinical research addressing the relevance and clinical outcomes of lateral augmentations (LA) as a whole [33, 49].

As a response to this controversy, a consensus was formulated on the anatomical description of the different elements of the ALC, along with the recognition of its role in the control of anterolateral subluxation of the knee [12]. The summary of recent biomechanical investigations observed that, except for minor differences between different types of reconstructions, the most common types of LA (e.g. ALL reconstruction, ITB based Lateral extraarticular Tenodesis (LET), Over-the-top ACLR with lateral augmentation) have the potential, in combination with intra-articular ACLR, to restore the kinematics of an ACL injured knee to those closer to that of a native knee joint [6, 11, 32, 51].

Even in the face of this biomechanical data, and despite good outcomes of the additional LA procedures being published in small case–control series with long-term follow-up [10, 17, 44, 64], the evidence to add a LA procedure to primary ACLR in order to improve patient outcomes has remained controversial. Given the more recent publications of high-quality clinical trials suggesting a reduction in anterolateral rotatory laxity and re-rupture rates of primary ACLR when combined with a LA [14, 55], we sought to determine whether the addition of a LA to a primary ACLR also ensures better objective knee stability scores and patient reported outcomes compared to an isolated ACLR. We hypothesized that an ACLR combined with any type of LA would result in superior objective knee stability examination and patient reported outcomes.

## Methods

### Search strategy

A literature search was performed based upon the guidelines of the Preferred Reporting Items for Systematic reviews and Meta-analyses (PRISMA) statement [35]. An electronic search including MEDLINE® and Embase® databases was conducted on February 6^th^, 2021 by two authors (LB and TV). The search query was compiled based upon a combination of following key words and MeSh terms ((*anterior cruciate ligament* OR *ACL*) AND *reconstruction* AND ((*anterolateral* AND (*ligament* OR *complex*)) OR (*lateral extra-articular tenodesis* OR *LET*) OR *iliotibial band tenodesis*) AND (*clinical* OR *functional* OR *failure* OR *outcome*)). The reference lists of included articles were carefully screened to identify additional eligible studies that were not retrieved by our electronic database search. All studies published from 2012 onwards were considered for inclusion in this systematic review if they met the eligibility criteria, as this was the year of publication of the early descriptions of the ALL.

### Eligibility criteria

#### Type of subjects

We included studies concerning patients with unilateral, isolated primary ACL injuries, indicated for a soft tissue ACLR with or without additional LA. Associated meniscal and osteochondral lesions in the ipsilateral knee, identified at the time of surgery with concomitant treatment, were no basis for exclusion. Studies were excluded based upon the use of synthetic grafts, both for the ACLR or the LA procedure, additional soft tissue procedures (ACL repair, multi-ligamentary reconstructions and meniscal transplant) or realignment procedures. Study specific inclusion and exclusion criteria of every eligible publication were listed, as well as extended indications for LA and separate failure criteria if reported (Table 1).

**Table 1:** Inclusion and exclusion criteria of included studies, with extended criteria for LA and description of accepted graft failure

Reference	Inclusion Criteria	Extra criteria for LA	Exlusion Criteria	Failure
	ACLR			
Castoldi et al. [1]	1. Complete isolated primary ACL rupture with a plan for arthroscopic ACLR, confirmed preoperatively by magnetic resonance imaging	NR	1. History of ACL repair or reconstruction 2. Associated tears of the posterior cruciate ligament 3. Injuries of the collateral ligaments requiring surgical treatment	Graft failure was defined by the presence of at least 1 of the following criteria: (1) Subsequent revision ACLR (2) Recurrent instability (> 1 episode) (3) A difference in anterior knee laxity (TELOS) > 10 mm (4) A soft endpoint in the Lachman test (5) A 3 + pivot-shift test (gross pivot shift) on physical examination
Ibrahim et al. [26]	Diagnosis of an unilatera ACL tear, confirmed by physical examination and magnetic imaging	Performed if one of following criteria were present: (1) Grade 2 pivot shift (2) High level of sporting activity (3) Participation in pivoting sports (4) Chronic ACL injury (5) Segond fracture	1. Revision ACL reconstruction 2. Multiligament knee injuries	NR
Hamido et al. [18]	1. Combined ACL rand ALL tears diagnose with MRI 2. Rupture high-grade pivot shift (III) 3. Segond fracture 4. A high level of sports activity 5. Participating in sports involving frequent pivoting	NR	1. History of knee surgery 2. History of knee dislocation 3. Preoperative signs of osteoarthritis 4. ACL revision surgery 5. Multiligamentous knee injury	Graft rupture
Sonnery-Cottet et al. [55]	1. All young patients (aged 16–30 years) 2. Participating in pivoting sports before injury 3. Decision to use a particular type of graft was based on patient factors/choice and the senior surgeon evolving indications for concomitant ALL reconstruction	1. Decision to use ALL reconstruction is based upon the senior surgeon evolving indications for concomitant ALL reconstruction	1. Collateral ligament injuries 2. Multiligament injuries 3. Undergoing other major concomitant procedures	Graft rupture
Goncharov et al. [15]	1. Workouts at least three times a week 2. Participation in competitions 3. Professional sports activities 4. Age from 16 to 40 years old 5. No previous surgical treatment of the study knee joint 6. Consent to MRI of the knee joint before the surgical treatment 7. No neurological and psychological disorders. 8. Consent to filling in the patient-reported outcomes and participation in the study	NR	NR	NR
Helito et al. [21]	1. More than 12 months since the injury, ACL lesion confirmed by clinical and imaging examinations 2. No peripheral ligament injuries apart from the anterolateral corner	NR	1. Procedures for axis correction 2. Treatment of chondral injuries 3. Meniscal repair or larger meniscectomies with resection of more than 50% of the meniscus width	Graft rupture, based on clinical instability and radiological criteria showing a new discontinuity of the graft
Helito et al. [22]	1. ACL injuries 2. Hyperlaxity patients based on the modified Beighton scale with evaluation of the contralateral limb to exclude any possible effects of trauma in the injured limb	NR	1. Collateral ligament injuries 2. Patients who had undergone previous surgery on the affected knee 3. Cases requiring axis correction by osteotomy 4. Patients with associated meniscal or chondral injuries requiring surgical treatment, except small meniscectomies (less than 50% of the meniscus width)	New ruptures defined on clinical ACL failure criteria:) 1. (physical examination showing laxity with no clear end point for Lachman and Anterior drawer tests (at least 2 + /3 +)) 2. (pivot-shift positivity (at least 2 + /3 +) associated with instability complaints) 3. (imaging showed a new graft rupture)
Rowan et al. [50]	1. ACL rupture 2. Radiological evidence of ACL rupture	Institutional indication for supplementary lateral extra-articular tenodesis is one major criterion or ≥ 2 minor criteria 1. Major: —High-grade pivot shift —Revision ACL reconstruction 2. Minor: —Hyperlaxity—Age < 20 years —Failed contralateral ACL reconstruction —Elite athlete	1. Concomitant repair or reconstruction of posterior cruciate ligament 2. Collateral ligament 3. Corner injuries 4. Undergoing revision ACL surgery	Re-injury of the reconstructed ACL
Porter et al. [45]	1. ACL rupture (diagnosed on MRI and at arthroscopic surgery) 2. Skeletally mature 3. Noncontact ACL injury 4. Involved in twisting/pivoting sports 5. ACL reconstruction performed within 6 weeks of injury 6. Pivot shift of at least 1 grade higher than contralateral knee after ACL reconstruction	NR	1. Other ligament injury greater than grade 1 or reparable meniscal tear 2. Previous ACL injury in either knee 3. Unwilling to be randomized to either treatment group 4. More than 6 weeks after ACL injury 5. Not fit for general anesthesia 6. Rheumatoid arthritis, connective tissue disease, or autoimmune disease	NR
Getgood et al. [14]	1. 14 and 25 years old 2. An ACL-deficient knee 3. Higher risk of reinjury based on the presence of 2 or more of the following factors: (1) Participation in competitive pivoting sports (2) Presence of a grade 2 pivot shift or greater (3) Generalized ligamentous laxity (Beighton score of 4 or greater) (4) Genu recurvatum greater than 10°	NR	1. Previous ACLR on either knee 2. Multiligament injury (> = 2 ligaments requiring surgical attention) 3. A symptomatic articular cartilage defect requiring treatment other than debridement 4. Greater than 3° of asymmetric varus 5. Unable or unwilling to be followed up for 2 years postoperatively 6. Skeletally immature	ACLR clinical failure with rotatory laxity defined as 1 or more following: (1) Persistent (detected at ≥ 2 visits) mild asymmetric pivot shift (grade 1) (2) A moderate or severe (grade 2 or 3) asymmetric pivot shift at any follow-up visit (3) A graft rupture, defined as a tear of the graft confirmed by either magnetic resonance imaging or arthroscopic examination
Vadalà et al. [58]	1. Presence of a moderate to severe rotatory instability as revealed by a pivot-shift test graded as + 2 or + 3 2. Minimum interval of two months between trauma and surgery and 3. Age less than 40 years old	1. Previous surgical procedures on the same or on the contralateral knee 2. Concomitant injury of the internal or the external collateral ligament 3. Concomitant systemic diseases 4. Pre-operative radiological signs of knee arthritis and imaging evidence of ICRS grades III or IV chondral damage on both patellar surface or medial and lateral femoral condyles	NR	NR

*ACL* Anterior Cruciate Ligament, *ACLR* Anterior Cruciate Ligament Reconstruction, *ALL* Anterolateral Ligament, *ICRS* International Cartilage Regeneration and Joint Preservation Society, *MRI* Magnetic Resonance Imaging, *NR* Not Reported, *TELOS* X-ray positioning knee holder

#### Type of interventions and comparisons

We aimed to compare isolated ACLR to ACLR combined with a LA procedure. All techniques of ACLR and LA procedure used were included, regardless the type of reconstruction (e.g. Single- or Double bundle), graft choice (e.g. Hamstrings, Quadriceps, Patellar tendon), graft fixation as well as the type and graft choice for LA (e.g. LET, ALL,…).

#### Type of outcome measurements

Objective stability scores and patient reported outcome scores were recorded (Table 2). Objective knee stability examination measurements included the Lachman and Pivot Shift tests. Instrumented laxity measurement with KT-1000 arthrometer was recorded where possible. Clinical failures were considered with a Lachman grade II or III and a Pivot Shift test grade II or III [14]. Patient reported outcomes comprised the International Knee Documentation Committee (IKDC) [19], Tegner Activity score [57] and Lysholm score [36]. Additionally, we obtained information on graft rupture rate and return to sports.

**Table 2:** Reported outcomes of the included studies in the systematic review

Ref	Patient reported outcomes					Objective knee examination				
	IKDC		Lysholm		Tegner	Lachman test	Pivot shift test	Arthrometer	Rerupture	Return to Play
	Continuous	Grading	Continuous	Grading						
Castoldi et al. [1]	✔		✔						✔	✔
Ibrahim et al. [26]		✔	✔	✔	✔	✔	✔	✔	✔	
Hamido et al. [18]		✔	✔	✔	✔	✔	✔	✔	✔	✔
Sonnery-Cottet et al. [55]	✔		✔		✔			✔	✔	✔
Goncharov et al. [15]	✔		✔							✔
Helito et al. [21]	✔		✔					✔	✔	
Helito et al. [22]	✔		✔				✔	✔	✔	
Rowan et al. [50]			✔		✔				✔	✔
Porter et al. [45]	✔		✔		✔		✔		✔	
Getgood et al. [14]	✔						✔		✔	
Vadalà et al. [58]	✔	✔	✔		✔	✔	✔	✔	✔	

*IKDC* International Knee Documentation Committee

#### Type of studies

We included all Level I-III studies reporting on the clinical outcomes of primary, isolated ACLR compared to ACLR combined with LA with at least 2 years of follow-up, comprising randomized controlled trials (RCTs) and prospective cohort studies without randomization. We excluded retrospective cohort studies and case series without a control group as well as systematic reviews, biomechanical and in-vitro studies, expert opinions, conference proceedings/abstracts and editorial comments, as well as publications written in any language other than English.

### Study selection

**Fig. 1:**
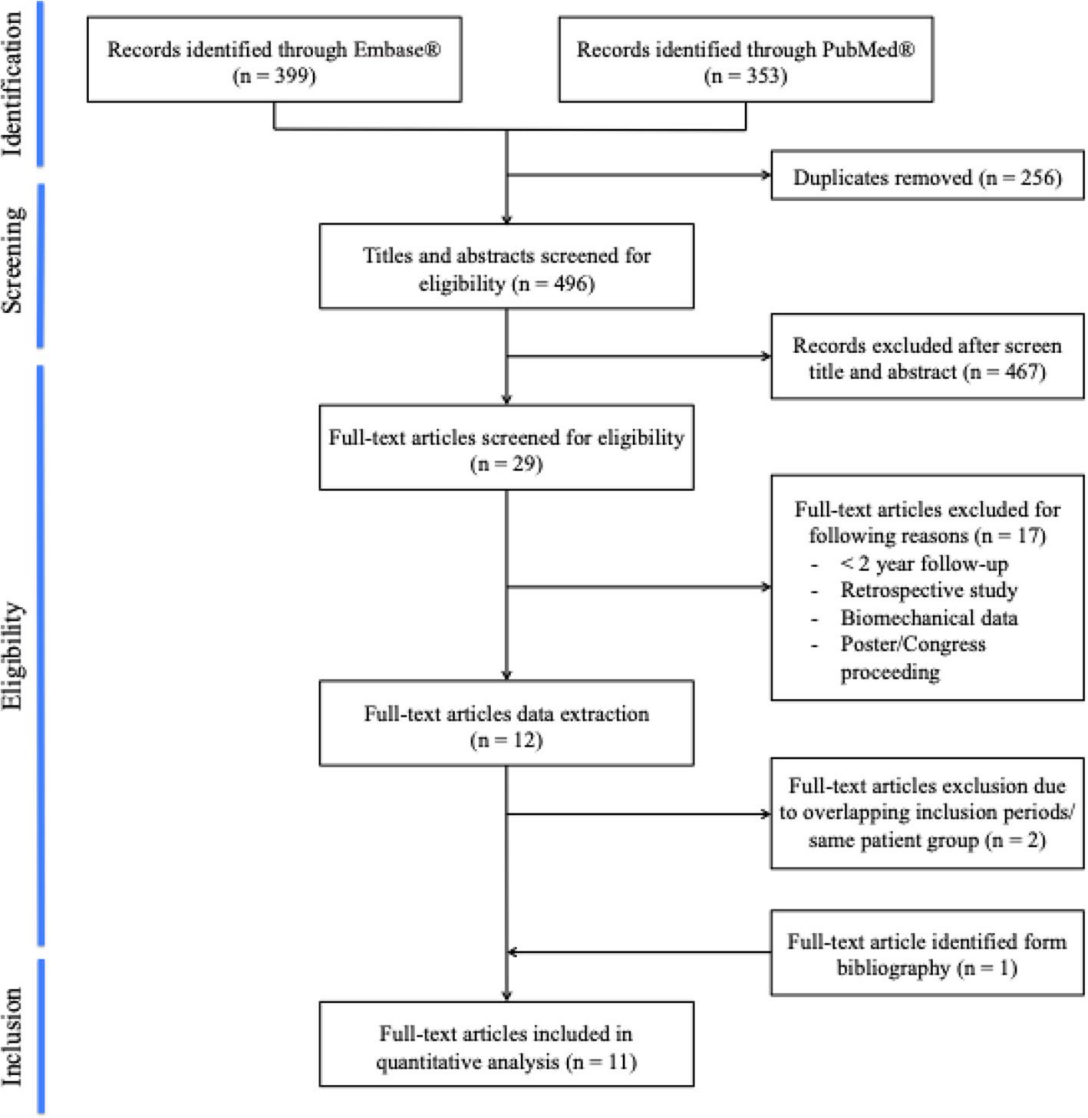
Flowchart of the followed study selection procedure according to the Preferred Reporting Items for Systematic reviews and Meta-Analyses (PRISMA) criteria

Two authors (LB and TV) independently screened the titles and abstracts of the identified studies obtained by the literature search and after removal of duplicate titles for their relevance (Fig. 1). All studies were considered for inclusion if they met the above stated inclusion criteria. A second, full text review was performed for the articles that passed the initial screening or in case of ambiguity in the title and abstract during the initial screening, unable to assess the eligibility of this publication on the limited information. In case of disagreement between the authors, the full text was reviewed conjointly and a decision was taken in consensus. A separate check of the reference lists of the included articles was performed to reveal publications that were initially missed during the literature search.

### Data extraction and quality assessment

Two authors (LB and TV) independently extracted study demographic data (Study design, Level of evidence, inclusion period, inclusion- and exclusion criteria, surgical techniques for ACLR and LA procedures, objective outcome data and patient reported outcomes graft rupture rate and return to sports). Risk-of-bias assessment was performed to evaluate the methodological quality of eligible studies by using the Cochrane Collaboration Tool for randomized controlled trials [24] and the Newcastle–Ottawa Scale (NOS) for the included prospective cohort studies [65].

### Statistical analysis

Statistical analyses and forest plots were performed using Cochrane Review Manager (version 5.3). Categorical outcomes were treated as dichotomous and the proportion of patients who had the event was determined. A pooled estimate of the overall odds ratio and 95% confidence interval (CI) was calculated using a Mantel–Haenszel test and random-effects model. For continuous outcomes, a pooled mean difference and 95% confidence interval were estimated using inverse weighting and a random-effects model. Standard deviation was estimated for studies that did not report a measure of variance according to the method described by Wan et al. [60]. We performed a sensitivity analysis to confirm that estimating variance did not significantly change the pooled treatment effect. The I^2^ statistic was used to assess between-study heterogeneity and was interpreted as low (25%), moderate (50%) or high (75%) according to Higgins and Thompson criterion [25]. We made a priori hypotheses that heterogeneity may be explained by ALL graft choices (gracilis/semitendinosus vs. IT band), study duration (< 3 years vs. > 3 years) or study design (RCT vs. cohort study). Statistical significance was set at *p *< 0.05.

## Results

### Systematic search and study selection

The initial literature search identified 752 studies (353 in Medline and 399 in Embase). After removal of duplicates, 496 studies remained and were subject to the first screening. Following review of title and abstract, 467 were excluded leaving 29 studies for full text review. Of those, 17 studies did not meet the inclusion criteria leaving 12 studies eligible for inclusion in this systematic review. During the data extraction, we found overlapping inclusion periods for six separate studies published by three different groups, reporting on similar outcome data with ambiguity as to whether all the included patients were unique [13, 14, 21, 22, 54, 55]. The subgroup analysis regarding concomitant medial meniscal repair in ACL reconstructions by Sonnery-Cottet et al. [54] shared a 17 month inclusion period (January 1, 2013 until May 31, 2014) with the previously published prospective cohort study [55]. This lead to an inevitable double patient inclusion as all patients with meniscal repair through a posteromedial portal were included. Therefore, this specific subgroup analysis was not included in this systematic review. Due to the lack of clarity regarding the patient groups published by Helito et al. [21, 22], the principal investigator was contacted and subsequently confirmed unique patient enrolment in both studies. Two studies published from the STABILITY 1 trail by Getgood et al. [13, 14] were also retrieved during the literature review. Both reported on graft re-rupture rate, but only patients and outcome data from the full RCT were retained for this systematic review [14]. Review of the included articles revealed one more article by Vadalà et al. eligible for inclusion [58]. Finally, 11 publications were assessed for systematic review and meta-analysis (Fig. 1).

### Characteristics of included studies

Following a thorough systematic review and data extraction 1892 unique patients were included in 11 studies. Of these patients, 1057 were treated with isolated ACLR and another 835 underwent ACLR with an additional LA (Table 3). Bone-Patellar Tendon-Bone (BPTB) grafts were used in two studies in both groups [1, 15], where as one study used the BPTB graft only in the isolated ACLR cohort [55]. All the other studies used Hamstrings Tendons (HT) as the ACL graft in both groups. In total, isolated ACLR were based on 177 (17%) BPTB and 880 (83%) HT grafts, while ACLR + LA relied on 56 (7%) BPTB and 779 (93%) HT grafts. Anterolateral Ligament Reconstruction (ALLR) was used as the LA in six studies (405 patients, 49%) [15, 18, 21, 22, 26, 55], although different reconstruction techniques were described and performed in these investigations. Three studies used an ITB based LET as an additional procedure (375 patients, 45%), again with different types of described techniques and grafts [1, 14, 50]. One publication described a Modified Iliotibial Band Tenodesis (28 patients, 3%) [45], as another study used a Cocker-Arnold (Modified Lemaire) procedure as their preferred LA technique (27 patients, 3%) [58]. Five publications were RCTs (925 unique patients, 465 ACLR and 460 ACLR + LA) [1, 14, 18, 26, 45] and six were prospective cohort studies (967 subjects, 592 ACLR and 375 ACLR + LA) [15, 21, 22, 50, 55, 58].

**Table 3:** Characteristics of the included studies in this systematic review

Reference	Type	LOE	Follow-up	Inclusion period				Knees at final FU			Mean Age (y)		Sex (M/F)		Surgical details			
				ACL		ACL + LA		All	ACL	ACL + LA	ACL	ACL + LA	ACL	ACL + LA	ACL	ACL + LA		
			(Mo)	Start	End	Start	End								Graft	ACL graft	AL type	AL graft
Castoldi et al. [1]	RCT	I	232.8	01/1998	09/1999	01/1998	09/1999	80	42	38	NR	NR	NR	NR	BPTB	BPTB	LET	Grac
Ibrahim et al. [26]	RCT	II	27	01/2014	06/2014	01/2014	06/2014	103	50	53	26	26	50/0	53/0	HT	HT	ALLR	Grac
Hamido et al. [18]	RCT	I	60	04/2014	03/2015	04/2014	03/2015	102	52	50	26	24	52/0	50/0	HT	HT	ALLR	Grac
Sonnery-Cottet et al. [55]	Cohort	II	38.4	01/2012	05/2014	01/2012	05/2014	502	176	221	23.5	21.8	116/60	152/69	HT	HT	ALLR	Grac
				01/2012	05/2014	-	-	-	105	-	22.1	-	96/9	-	BPTB	-	-	-
Goncharov et al. [15]	Cohort	II	24	2014	2015	2014	2015	48	30	18	NR	NR	NR	NR	BPTB	BPTB	ALLR	Grac/SemiT
Helito et al. [21]	Cohort	III	26	01/2011	06/2012	01/2014	06/2015	101	68	33	33.9	33.1	59/9	30/03	HT	HT	ALLR	Grac
Helito et al. [22]	Cohort	III	28.1	01/2011	01/2013	01/2015	08/2016	90	60	30	29.9	27	28/32	13/17	HT	HT	ALLR	Grac
Rowan et al. [50]	Cohort	III	27	NR	NR	NR	NR	171	125	46	29	27	67/58	27/19	HT	HT	LET	ITB
Porter et al. [45]	RCT	II	24	07/2014	01/2017	07/2014	01/2017	51	23	28	22.3	21.8	NR	NR	HT	HT	MITBT	ITB
Getgood et al. [14]	RCT	I	24	01/2014	03/2017	01/2014	03/2017	589	298	291	18.8	19.1	NR	NR	HT	HT	LET	ITB
Vadalà et al. [58]	Cohort	II	44.6	01/2005	12/2006	01/2005	12/2006	55	28	27	28	26	NR	NR	HT	HT	CA	ITB

*ACL* Anterior Cruciate Ligament, *ALLR* Anter Lateral Ligament Reconstruction, *BPTB* Bone-Patellar Tendon-Bone, *CA* Cocker-Arnold (Modified Lemaire procedure), *Grac* Gracilis, *HT* Hamstrings Tendon, *ITB* IlioTibial Band, *LA* Lateral Augmentation, *LET* Lateral Extraarticular Tenodesis, *LOE* Level of Evidenc, *MITBT* Modified Iliotibial Band Tenodesis, *NR* Not Reported, *RCT* Randomized Control Trial, *SemiT* Semitendinosus

### Risk of bias

The lateral skin incision makes it impossible to blind the patients for an extra LA procedure, inducing a performance bias risk in all RCTs [1, 14, 18, 26, 45]. By implementing the Cochrane Collaboration Tool, we identified extra high risks on bias in publications by Castoldi et al. due to block randomization [1] and Ibrahim et al. as a result of allocation and random sequence generation based upon the date of birth of the subjects [26] (Table 4). Assessment of the prospective cohort studies [15, 21, 22, 50, 55, 58] using the NOS scoring system demonstrated good quality for all the included publications (Table 5).

**Table 4:** Risk-of-bias assessment of the included Randomized Control Trails using the Cochrane Collaboration Tool

Reference	Random sequence generation	Allocation concealment	Selective reporting	Other sources of bias	Blinding (participants and personnel)	Blinding (outcome assessment)	Incomplete outcome data
Castoldi et al. [1]	High Risk	Low Risk	Unclear	Unclear	High Risk	Low Risk	Low Risk
Ibrahim et al. [26]	High Risk	High Risk	Low Risk	Unclear	High Risk	Low Risk	Low Risk
Hamido et al. [18]	Low Risk	Low Risk	Low Risk	Unclear	High Risk	Low Risk	Low Risk
Porter et al. [45]	Low Risk	Low Risk	Low Risk	Unclear	High Risk	Low Risk	Low Risk
Getgood et al. [14]	Low Risk	Low Risk	Low Risk	Unclear	High Risk	Low Risk	Low Risk

**Table 5:** Risk-of-bias assessment of the included prospective cohort studies using the Newcastle–Ottawa Quality assessment Scale

Reference	Selection				Comparability	Outcome			Overall Quality
	Representativeness of the exposed cohort	Selection of the non-exposed cohort	Ascertainment of exposure	Demonstration that outcome of interest was not present at start of study	Comparability of cohorts on the basis of the design or analysis controlled for confounders	Assessment of outcome	Was follow-up long enough for outcomes to occur	Adequacy of follow-up of cohorts	
Sonnery-Cottet et al. [55]	*	*	*	*	*	*	*	*	Good
Goncharov et al. [15]	/	*	*	*	*	*	*	*	Good
Helito et al. [21]	*	*	*	*	*	*	*	*	Good
Helito et al. [22]	*	*	*	*	*	*	*	*	Good
Rowan et al. [50]	*	*	*	*	*	*	*	*	Good
Vadalà et al. [58]	*	*	*	*	*	*	*	*	Good

*: criteria met, / Criteria not met or unable to determine

Good quality: 3 or 4 stars in selection domain AND 1 or 2 stars in comparability domain AND 2 or 3 stars in outcome/exposure domain

Fair quality: 2 stars in selection domain AND 1 or 2 stars in comparability domain AND 2 or 3 stars in outcome/exposure domain

Poor quality: 0 or 1 star in selection domain OR 0 stars in comparability domain OR 0 or 1 stars in outcome/exposure domain

### Patient reported outcome scores

**Fig. 2:**
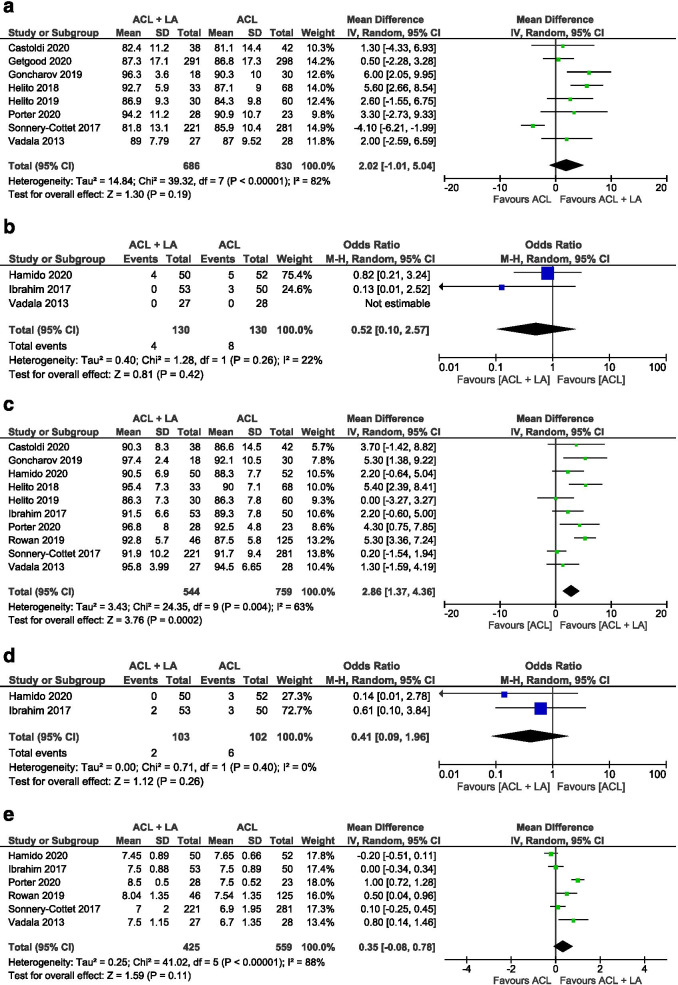
Forest plots of patient-reported outcomes scores (Mean difference/Odds ratio and 95% CI) of a IKDC score (reported as continuous data) b IKDC score (reported as 4-Grade scale scoring system) c Lysholm score (reported as continuous data) d Lysholm score (reported as 4-Grade scale scoring system) e Tegner score (CI, confidence interval; IV, inverse variance statistical method; M-H, Mantel–Haenszel statistical method)

*IKDC*: The IKDC score was reported by 10 publications (eight studies mentioned the score as continuous data [1, 14, 15, 21, 22, 45, 55, 58] with three studies making use of the 4-grade scale [18, 26, 58]. No significant difference was observed in the final IKDC scores between the isolated ACLR and the ACLR + LA procedures (Continuous data: mean difference 2.02, 95% CI -1.01 to 5.04, I^2^ = 82%, *p *= 0.19 and 4-Grade scale scoring system: OR 0.51, 95% CI 0.16 to 1.67, I^2^ = 22%, *p *= 0.27) (Fig. 2a-b). Of note is the high observed heterogeneity in the continuous IKDC data.

*Lysholm score*: The postoperative Lysholm score at final follow-up was recorded in 10 publications [1, 15, 18, 21, 22, 26, 45, 50, 55, 58], with two reporting both continuous data and graded results [18, 26]. Three Studies reported the Lysholm score as median and interquartile rage (IQR) data [18, 26, 50] while seven studies mentioned results as Mean +—standard deviation (SD) [1, 15, 21, 22, 45, 55, 58]. The subjects treated with a combined ACLR and LA had significantly better knee function scores compared to those who underwent treatment with isolated ACLR (Continuous data: mean difference 2.86, 95% CI 1.37 to 4.36, I^2^ = 63%, *p *< 0.001 and 4-Grade scale scoring system: OR 0.41, 95% CI 0.09 to 1.96, I^2^ = 0%, *p *= 0.26) (Fig. 2c-d). Sensitivity analysis showed no difference for the estimated mean and variance of all the articles compared to those specifically reporting mean +—SD (mean difference 2.61, 95% CI 0.71 to 4.51).

*Tegner*: Six studies reported on the Tegner activity score, including four RCTs and two prospective cohort studies [18, 26, 45, 50, 55, 58]. Three studies reported median and IQR data [18, 26, 45], while three others used mean and SD [50, 55, 58]. No significant difference could be found in the Tegner score between patients treated with an isolated ACLR reported and those treated with a combined procedure (mean difference 0.35, 95% CI -0.08 to 0.78, I^2^ = 88%, *p *= 0.11) (Fig. 2e). No difference was found between the complete group and the subgroup reporting with mean +—SD upon sensitivity analysis (mean difference 0.28 (95% CI -0.17 to 0.75).

### Graft rupture

**Fig. 3:**
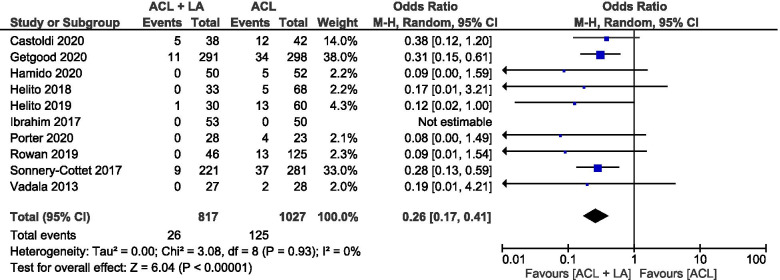
Forest plot of graft rupture rate (Odds ratio and 95% CI) (CI, confidence interval; M-H, Mantel–Haenszel statistical method)

Graft rupture rate was reported in 10 studies [1, 14, 18, 21, 22, 26, 45, 50, 55, 58], with Ibrahim et al. reporting no re-ruptures in both groups [26]. The overall graft rupture rate was significantly lower in the ACLR + LA group (3%) than the isolated ACLR group (12%) (OR 0.26, 95% CI 0.17 to 0.41, I^2^ = 0%, *p *< 0.001) (Fig. 3).

### Objective knee stability examinations

**Fig. 4:**
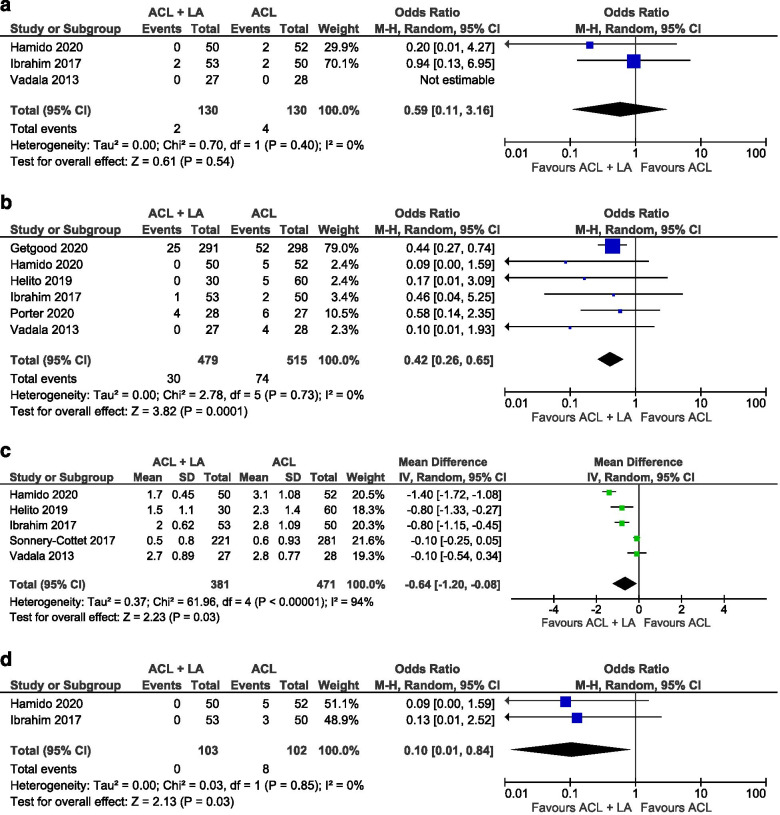
Forest plots of clinical outcomes (Mean difference/Odds ratio and 95% CI) of a Lachman test b Pivot shift test c Arthrometer (KT-1000, reported as continuous Side-to-side difference) d Arthrometer (KT-1000, reported as stage Side-to-side difference) (CI, confidence interval; IV, inverse variance statistical method; M-H, Mantel–Haenszel statistical method)

*Lachman Test*: The Lachman test was reported in three studies (two RCTs and one cohort study) [18, 26, 58] and reviewed in 260 knees. The frequency of negative graded tests from the included patients treated with an isolated ACLR (84%) was not significant from those treated with a combined procedure (87%). (OR 0.59, 95% CI 0.11 to 3.16, I^2^ = 0%, *p *= 0.54) (Fig. 4a).

*Pivot shift test*: A Pivot Shift test was reported in six studies [14, 18, 22, 26, 45, 58] including 994 knees. The results at final follow-up of the Pivot Shift test from the STABILITY trail [14] were included after contact with the first author, as they weren’t separately mentioned in the publication. The frequency of positive graded tests (grade II and III) was 6% in the group of patients treated with an ACLR and LA and 14% in the group of patients who underwent an isolated ACLR. (OR 0.42, 95% CI 0.26 to 0.65, I^2^ = 0%, *p *> 0.001) (Fig. 4b).

*Instrumented laxity (KT-1000 Arthrometer)*: Side-to-side anterior translation differences, quantified by KT-1000 Arthrometer measurements, were recorded in six publications [18, 21, 22, 26, 55, 58], including a total of 852 knees. Three reported the difference as median and IQR data [18, 21, 26] (two of them in combination with graded data [18, 26]) and three other studies as Mean +—standard deviation [22, 55, 58]. Significant differences in the instrumented anterior translation was found between isolated ACLR group and the combined reconstruction group (mean difference -0.64, 95% CI -1.20 to -0.08, I^2^ = 94%, *p *= 0.03) (OR 0.10, 95% CI 0.01 to 0.84, I^2^ = 0%, *p *= 0.03) (Fig. 4c-d). Sensitivity analysis didn’t revealed differences when reporting as a whole group compared to subgroup of studies reporting with mean +—SD subgroup (mean difference –0.64 (95% CI -1.20 to -0.08).

### Return to play

**Fig. 5:**
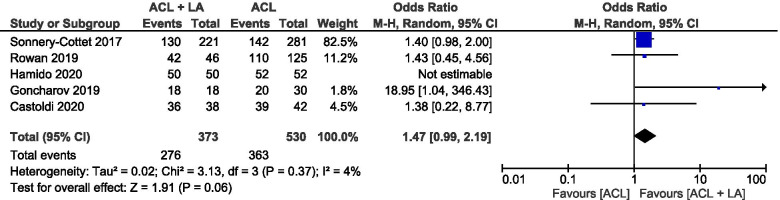
Forest plot of return to sports (Odds ratio and 95% CI) (CI, confidence interval; M-H, Mantel–Haenszel statistical method)

Return to the same level of play was recorded in five publications [1, 15, 18, 50, 55]. No significant difference was noted in the return to play between the patients who underwent an isolated ACLR (68%) and those who were treated with an ACLR + LA (74%) (mean difference 1.47, 95% CI 0.99 to 2.19, I^2^ = 4%, *p *= 0.06). (Fig. 5). Of note, one study, which included only male athletes, reported a 100% return to sports in both groups [18].

## Discussion

The most important finding of our systematic review is that the addition of a LA to a primary ACLR results in significant reductions in graft failure and persistent rotatory laxity at a minimum of two years post operatively. The identification of generally superior patient reported outcome scores and a higher proportion of return to sport in patients treated with an ACLR + LA adds further weight to the argument that contemporary LA techniques should be considered when treating ACL injured patients who are deemed at high risk of graft failure.

Our hypothesis of ACL + LA procedures providing superior objective and clinical outcomes is generally supported, particularly in regard to rotational stability testing, as determined by the Pivot Shift test. Clinical and biomechanical insights have evolved over the past decade regarding the ALC as a rotatory stabilizer in ACL deficient and reconstructed knees. Ferretti et al. described that in up to 90% of ACL injured knees, additional lesions were found to the lateral structures [9]. Inferior clinical results were noted by Sobrado et al. when comparing patients with ACL reconstructed knees and concomitant, but untreated ALL lesions to patients treated for isolated ACL ruptures with intact lateral structures [52]. These clinical studies are supported by overwhelming biomechanical data regarding the role of the ALC as a more efficient lever arm to control the rotatory translation when compared to an isolated ACLR [11, 32, 39, 56]. Subsequently, several philosophies and techniques have emerged over the past decade in an attempt to restore the anatomy and/or function of the ALC.

Reconstruction of the ALL, a fibrous band in the anterolateral capsule initially identified by Segond, has been described in a number of different forms. These aim to be as anatomic as possible; however, different descriptions of the anatomy of the ALL has led to a variation in ALL graft insertion points, particularly in relation to its tibial insertion most recently. The original technique developed by Claes utilised a single graft coursing anterior and distal to the lateral collateral ligament femoral insertion to a position midway between the fibula head and Gerdy’s tubercle on the tibia. Later single graft procedures popularised by Helito et al. [20] have been revised to a more posterior and proximal position on the femur with a similar tibial insertion to obtain the functional anisometry in the ALLR graft [27]. The reconstruction developed by Sonnery-Cottet et al. [53] has used the same femoral origin but uses a wider footprint insertion on the tibia creating a double graft structure tensioned in extension. Even with these variations in technique, the results seem to speak for themselves. The addition of the ALLR seems to reduce rotatory laxity and graft failure.

An alternative approach in the effort to improve the rotational stability of intra-articular ACLR, are the different types of modified LETs, derived from abandoned ‘historical’ isolated extra-articular tenodeses [51]. The common feature of these techniques is the addition of a lateral soft tissue restraint on a certain distance from the central pivot of the knee [30]. Unlike the ALLR, these non-anatomical reconstructions are roughly isometric throughout the range of motion, aiming to restore the function of the several lateral structures of the ALC that are involved in the rotatory stabilization of the knee [11, 32, 41].

Although on-going controversy remains if a specific type of LA is superior [47], the results of our systematic review demonstrate that adding either an ALL reconstruction or LET procedure significantly improves the rotatory stability, which is consistent with previous published systematic reviews and meta-analyses [4, 7, 23, 48].

The pooled data for the anterior stability tests did not show a significant difference between isolated or combined ACLR procedures when performed manually with the Lachman Test. However, the addition of the LA procedure appears to limit the extreme antero-posterior translation, as observed with the significant reduction in the side-to-side differences measured with the KT-1000 Arthrometer testing. This may indicate that an isolated ACLR is able to control antero-posterior translation and maybe sufficient in the treatment of ACL deficient knees when significant rotator laxity is not present. However, this also may point to the potential benefit of LA in reducing ACL graft strain as seen in a cadaveric studies by Engebretsen et al. [8] and more recently by Marom et al. [37], identifying a significant reduction in graft forces when a LET type augmentation was added to an ACLR. This may also account for the significant reduction in graft re-rupture rates in the combined group that were observed. Possible explanations are the superior rotational stability with the added LA, but also by the perceived load-sharing effect of a LA in combination of an intra-articular ACLR. Adding a LA might reduce the deformity of the graft during the early ligamentization process, promoting final graft strength and subsequent reduced graft failure [44]. This is supported by a recent publication by Cavaignac et al., identifying better maturation and incorporation of 4-strand hamstrings ACL grafts at the 1 year interval when combined with a LA as observed on MRI [3].

Some discrepancy exists regarding the patient reported outcome scores, and more specifically concerning the dedicated knee scores. No significant differences could be found between the ACLR and ACLR + LA groups when reviewing the IKDC scores. Conversely, the Lysholm score showed a significant improvement in the combined treated group. This may suggest that the Lysholm score could be better at picking up differences in outcomes specifically related to rotatory laxity. However, it is challenging to draw conclusions due to the significant heterogeneity that was observed when pooling the IKDC and Lysholm scores, similar to previous published systematic reviews [4, 23, 62]. Possible explanations for this are the high variability between studies regarding type of ACLR and LA procedures as well as the included patient characteristics. On the other hand, Xu et al. [62] reported similar heterogeneity although their systematic review included only ALL reconstructions, indicating a possible inherent effect of the scoring system on these results. No significant differences were observed between the two groups regarding the activity related Tegner score.

One study identified an initial delay in the recovery in the ACLR + LA combined groups due to a higher amount of pain along with a delayed recovery in quadriceps strength, resulting in initial reduced subjective outcomes when compared to isolated ACLR patients [14]. This delay was attributed to the additional lateral procedure but proved to be transient as the differences resolved by the 6 months postoperative review [13]. Our results indicate that at minimum 2 years follow up, patients treated with a combined procedure have equivalent to superior outcomes, which is consistent with recent systematic reviews [4, 62], but deviates from older reviews [7, 23, 48]. Possible explanations for these superior results are the improved knee rotatory stability with the newer, more dedicated LA procedures along with the observed equivalent isokinetic muscle recovery in patients treated with ACLR + LA [13, 28]. These findings are also likely the reason of the higher, although not significant, degree of return to sports observed in the ACLR + LA treated group. After completing full rehabilitation, better objective rotational stability and subjective functional outcomes tend to promote a higher return to sports.

Our systematic review is characterized by a number of limitations, which must be considered when interpreting the findings. First, the specific inclusion of all types of ACLR and LA allows for great variability in surgical techniques and graft choices. The inclusion of different patient populations, indications, and differing treatment of concomitant meniscal and cartilage lesions as well as lack of standardised post-operative rehabilitation, may also create a significant selection bias. However, this also speaks to the generalizability of the findings to a wider patient population. Furthermore, it was the author’s intention to include any type of LA, as we wanted to evaluate the clinical effect of an additional lateral procedure in ACLR, independent from their different surgical techniques. Second, our choice to include only studies from 2012 onwards seems arbitrary but is based on the LA’s renaissance with the 'rediscovery' of the ALL. New techniques and surgical indications have emerged since these publications. By choosing this date, we intended to include studies that would be influenced by these new insights utilising contemporary techniques in current clinical practice. Unfortunately, choosing this restricted inclusion time period meant that some long-term follow-up studies by surgeons who were early advocates of the concept of a LA procedure, are not included in this systematic review [10, 38, 63]. Finally, we did not address the possible adverse events and consequences of an additional LA procedure, as only a limited number of studies reported on specific issues related to the LA [14, 18, 55]. Only one, underpowered study mentioned long-term radiographic follow-up for lateral tibiofemoral osteoarthritis [1].

Even in the face of this compelling data, routine implementation of any type of LA in primary ACLR remains controversial as insights continue to evolve around the indications and surgical techniques. Several high-level RCTs regarding LA procedures with different types of intra-articular grafts are highly anticipated (Stability 2 (ClinicalTrails.gov identifier NCT03935750), SANTI RCT (NCT03740022)) and might provide further insights on the indications for LA procedures. For now, young age (< 25), return to pivoting contact sports and the use of a hamstring autograft are indications for LA of primary ACLR in our practice. The presence of knee hyperextension, meniscal deficiency and increased tibial slope, even when using other grafts such as bone patella tendon bone, are considerations for the addition of a LA. Due to the known inferior results associated with revision ACLR, the majority of revisions are augmented by an LET in our practice.

New prospective research will need to focus on the identification of patients at risk of inferior results and higher re-rupture rates when treated with an isolated ACLR. Further individualisation of the treatment approach will be necessary to optimize patient important outcomes.

## Conclusion

Conflicting anatomic and biomechanical data surrounding the ALC, amplified by differences of opinion in the surgical community, has led to controversy surrounding the use of LA procedures in primary ACLR. Based upon this systematic review of contemporary clinical literature, and findings from previously performed systematic reviews [4, 7, 23, 48, 62], the addition of a LA to primary ACLR can significantly reduce the risk of graft rupture and persistent rotatory laxity, without jeopardizing patient reported outcomes. Future research will focus on when to add these procedures, not if.

## Data Availability

Data obtained during the literature review by the independent authors are available upon request.

## Abbreviations

ACL: Anterior cruciate ligament
ACLR: Anterior cruciate ligament reconstruction
ALC: Anterolateral complex
ALL: Anterolateral ligament
ALLR: Anterolateral ligament reconstruction
BPTB: Bone-patellar tendon-bone
CI: Confidence interval
HT: Hamstrings tendon
ITB: Iliotibial band
IQR: Interquartile range
IV: Inverse variance (statistical method)
LA: Lateral augmentation
LET: Lateral extra-articular tenodesis
M-H: Mantel–Haenszel (statistical method)
OR: Odds ratio
NOS: Newcastle–Ottawa Scale
PRISMA: Preferred reporting items for systematic reviews and meta-analyses
RCT: Randomised Control Trail
SD: Standard deviation
